# Acute liver failure and hemolytic anemia induced by quetiapine and aripiprazole overdose in a patient with schizophrenia and metastatic breast cancer: a unique case report

**DOI:** 10.3389/fpsyt.2026.1782761

**Published:** 2026-04-13

**Authors:** Hua Zhong, Shu Zhou

**Affiliations:** 1 Emergency Department, Liuyang People’s Hospital, Liuyang, Hunan, China; 2 Emergency Department, Liuyang People’s Hospital, Changsha, Hunan, China

**Keywords:** acute liver failure, aripiprazole, drug toxicity, hemolytic anemia, quetiapine

## Abstract

This case report describes a rare situation in which a patient with schizophrenia and metastatic breast cancer experienced acute liver failure and hemolytic anemia caused by an overdose of quetiapine and aripiprazole. On the day of admission, the patient received lipid emulsion infusion, continuous renal replacement therapy (CRRT), and blood perfusion. After these treatments, the patient’s consciousness improved from mild coma to full awareness. However, 48 hours after admission, the patient developed hemolytic anemia and acute liver failure. Following supportive treatments like plasma exchange, bilirubin adsorption, washed red blood cell transfusion, and low-dose dexamethasone for inflammation, the patient recovered and was discharged. This is the first reported case of hemolytic anemia and acute liver failure caused by mixed toxicity of quetiapine and aripiprazole in an adult patient. We analyze the characteristics of this case to enhance awareness of toxicity from atypical antipsychotics like quetiapine and aripiprazole, and to heighten vigilance regarding the potential risks of combined medication in patients with underlying liver disease, thereby improving the success rate of treatment.

## 1 Introduction

Acute liver failure is characterized by sudden liver dysfunction, jaundice, coagulopathy, and multi-organ failure. It can be triggered by various causes, including viral hepatitis, autoimmune liver disease, and drug-induced liver injury. In the United States, drug-induced liver injury is the leading cause of acute liver failure, accounting for approximately 2-5% of hospitalized patients with jaundice (1). Pathologically, it can be classified into four major injury patterns: hepatocellular injury, cholestasis, mixed injury, or fatty degeneration (2).

Both quetiapine and aripiprazole are atypical antipsychotics primarily metabolized by the liver. Aripiprazole acts as a partial agonist at dopamine (DA) D2 and 5-hydroxytryptamine (5-HT) 1A receptors, while antagonizing 5-HT2A receptors; this modulation helps maintain neurotransmitter stability (3). Quetiapine blocks D2/5-HT receptors and interacts with various neurotransmitter receptors. It shows high affinity for 5-HT2A, α1, and H1 receptors; moderate affinity for D2 and α2 receptors; and lower affinity for D1 and M1 receptors (4). This profile effectively controls both positive and negative symptoms of psychosis. A report by Sidi He et al. in 2024 analyzed 408 cases of drug-induced liver injury caused by antipsychotics extracted from the U.S. Food and Drug.

Administration (FDA) Adverse Event Reporting System (FAERS) database, concluding that the use of all atypical antipsychotics was not significantly associated with an increased risk of hepatotoxicity (5). However, there have been reports of two cases of liver failure solely due to quetiapine (6, 7). Compared to quetiapine, aripiprazole is less likely to cause liver injury, and patients’ liver function tends to recover more easily after injury. Here, we report a case of acute liver failure and hemolytic anemia caused by mixed drug toxicity of quetiapine and aripiprazole. This case was successfully treated at our hospital.

## 2 Case introduction

### 2.1 Patient information

The patient is a 53-year-old male, of Han ethnicity, and a farmer.

### 2.2 Clinical symptoms

At approximately 14:00 on July 25, 2025, the patient was found by family members to be confused and slurring his speech. Subsequently, his consciousness disturbance worsened to the point of being unresponsive to calls, presenting in a state of light coma. He was urgently transported to the hospital by ambulance.

### 2.3 Past medical history

The patient has a history of schizophrenia diagnosed by an external psychiatric department for over 10 years. He intermittently took oral Aripiprazole Orally Disintegrating Tablets 10mg/d and Quetiapine Fumarate Tablets 50mg/d to control the condition, with irregular outpatient follow-ups. During this hospitalization, a psychiatric examination by a psychiatrist indicated the presence of persecutory delusions, decreased volitional activity, drug-swallowing suicidal behavior, and a lack of insight. The patient discovered a mass on the left chest wall over 2 years ago but refused diagnosis and treatment due to personal reasons. He denies other medical histories such as hypertension, diabetes, hepatitis, or tuberculosis. No history of smoking or alcohol consumption.

### 2.4 Physical examination

Body temperature 36.6 °C, pulse 123 beats/minute, respiratory rate 22 breaths/minute, blood pressure 124/82 mmHg. The patient was in a state of light coma, with a Glasgow Coma Scale (GCS) score of E1V1M4 = 6. Both pupils were equal in size and round, with a diameter of approximately 2 mm, and showed sluggish light reflex. The neck was supple. A large mass measuring about 8cm × 8cm was palpable on the left chest wall centered around the nipple, with local skin showing redness, swelling, ulceration, and pus discharge. The rest of the skin and mucous membranes were normal. Enlarged lymph nodes were palpable in the left axilla. Bilateral lung breath sounds were coarse, with audible moist rales. Muscle tone in the limbs was mildly increased, with no other significant positive signs.

### 2.5 Emergency auxiliary tests and main diagnosis

Head computed tomography (CT) and diffusion-weighted imaging (DWI) ruled out cerebrovascular accident. Head computed tomography (CT) and diffusion-weighted imaging (DWI) ruled out cerebrovascular accident. White blood cell count 13.65 × 10^9^/L (normal reference range: 3.5–9.5 × 10^9^/L), neutrophil count 12.42 × 10^9^/L (normal reference range: 1.8–6.3 × 10^9^/L), platelets 229 × 10^9^/L (normal reference range: 125–350 × 10^9^/L), hemoglobin 126 g/L (normal reference range: 130–175 g/L). Liver function tests showed: Alanine aminotransferase (ALT) 54 U/L (normal reference range: 9–50 U/L), Aspartate aminotransferase (AST) 85 U/L (normal reference range: 15–40 U/L), total bilirubin (TBIL) 30.6 μmol/L (normal reference range: 0–26 μmol/L), direct bilirubin (DBIL) 15.6 μmol/L (normal reference range: 0–8 μmol/L), total bile acid (TBA) 2.8 μmol/L (normal reference range: 0–6.71 μmol/L), indirect bilirubin (IBIL) 15.0 μmol/L (normal reference range: 3.4–17 μmol/L). Toxicological testing confirmed the presence of quetiapine and aripiprazole in urine, with blood concentrations of quetiapine 6687.1 ng/mL and aripiprazole 187.3 ng/mL, respectively, confirming a diagnosis of mixed drug poisoning.

### 2.6 Treatment process

The patient was admitted beyond the time window for gastric lavage. Long-chain lipid emulsion was infused to reduce the volume of drug distribution. Blood purification (CVVHDF + HP) was initiated 4 hours after admission and continued for 10 hours using two HA330 resin perfusion devices. Supportive treatments included activated charcoal to absorb toxins, as well as hydration and diuresis to promote toxin excretion. Other measures focused on gastric protection, nebulized expectoration, human albumin supplementation, correction of acid-base balance, maintenance of water and electrolyte balance, and administration of norepinephrine to maintain blood pressure. Due to significant oral secretions and some food residue, along with altered consciousness and lung CT findings, aspiration pneumonia was suspected, and piperacillin-tazobactam was administered for infection control.13 hours after treatment, the patient was fully conscious and admitted to taking approximately 0.1g of quetiapine (42 tablets) and 5mg of aripiprazole (20–30 tablets) about 7 hours before admission. However, the patient refused bronchoscopy, tumor biopsy, and any tumor-related treatment. At this point, renal function normalized, liver function markers decreased to near normal, and coagulation tests showed mild abnormalities. Cardiac enzyme levels, white blood cell counts, and neutrophil counts all decreased compared to previous values. On the third day after admission, the patient suddenly developed acute liver failure, evidenced by a significant increase in bilirubin and lactate dehydrogenase (LDH), elevated blood urea nitrogen, decreased hemoglobin, and mild elevation of liver transaminases. Inflammatory markers slowly increased, whereas creatinine levels remained stable and within the normal range.

**Figure 1:**
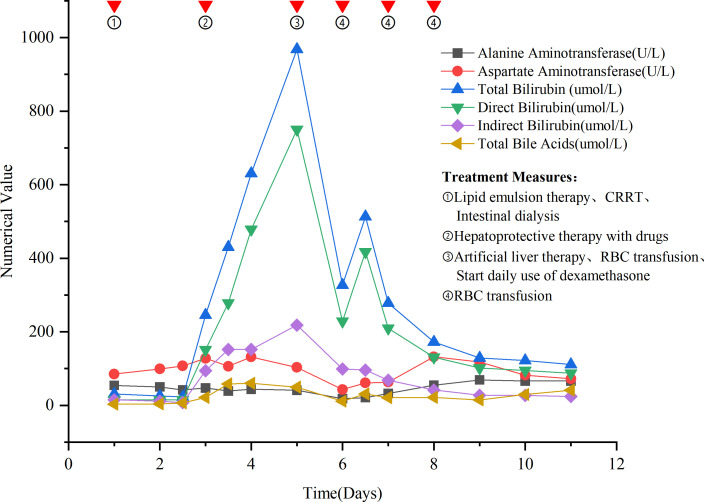
Changes in liver function injury markers and treatment measures.

During hospitalization, the values and trends of liver function injury markers are shown in **Figure 1**.

**Figure 2:**
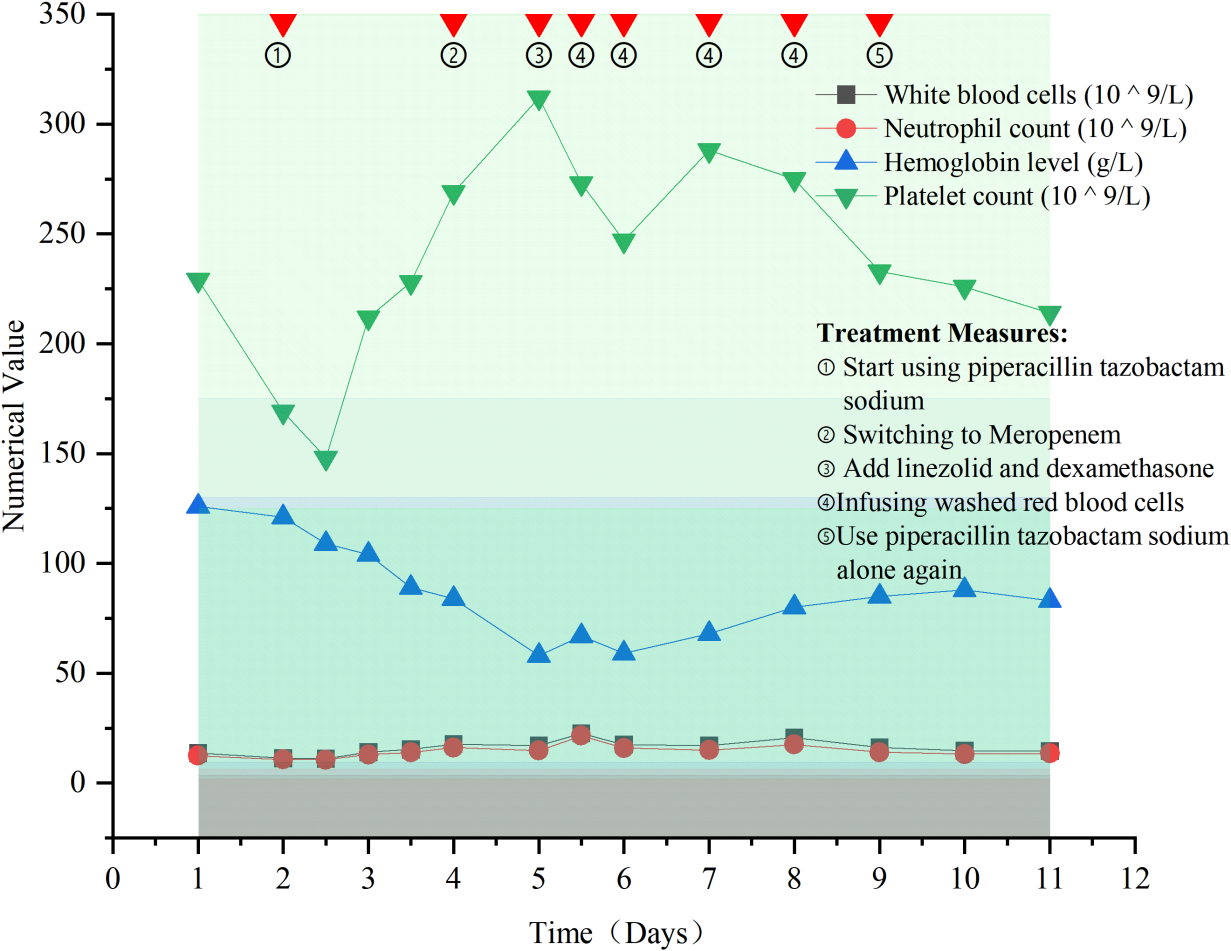
Trends in blood cell values and treatment measures.

The values and trends of blood cell analysis indicators are shown in **Figure 2**.

**Figure 3:**
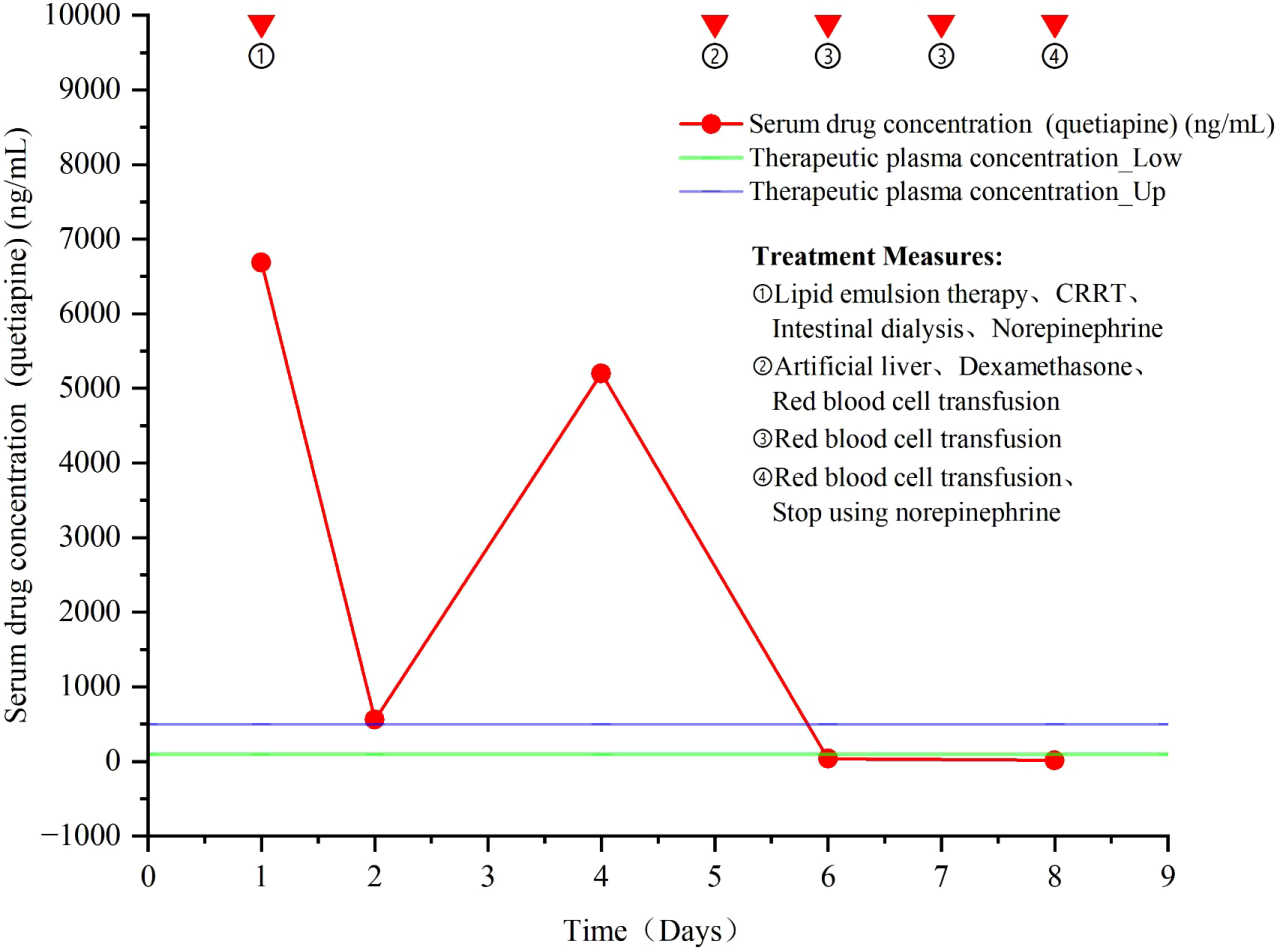
Change trend of quetiapine drug concentration and treatment measures.

The trend of quetiapine blood concentration is shown in **Figure 3**.

**Figure 4:**
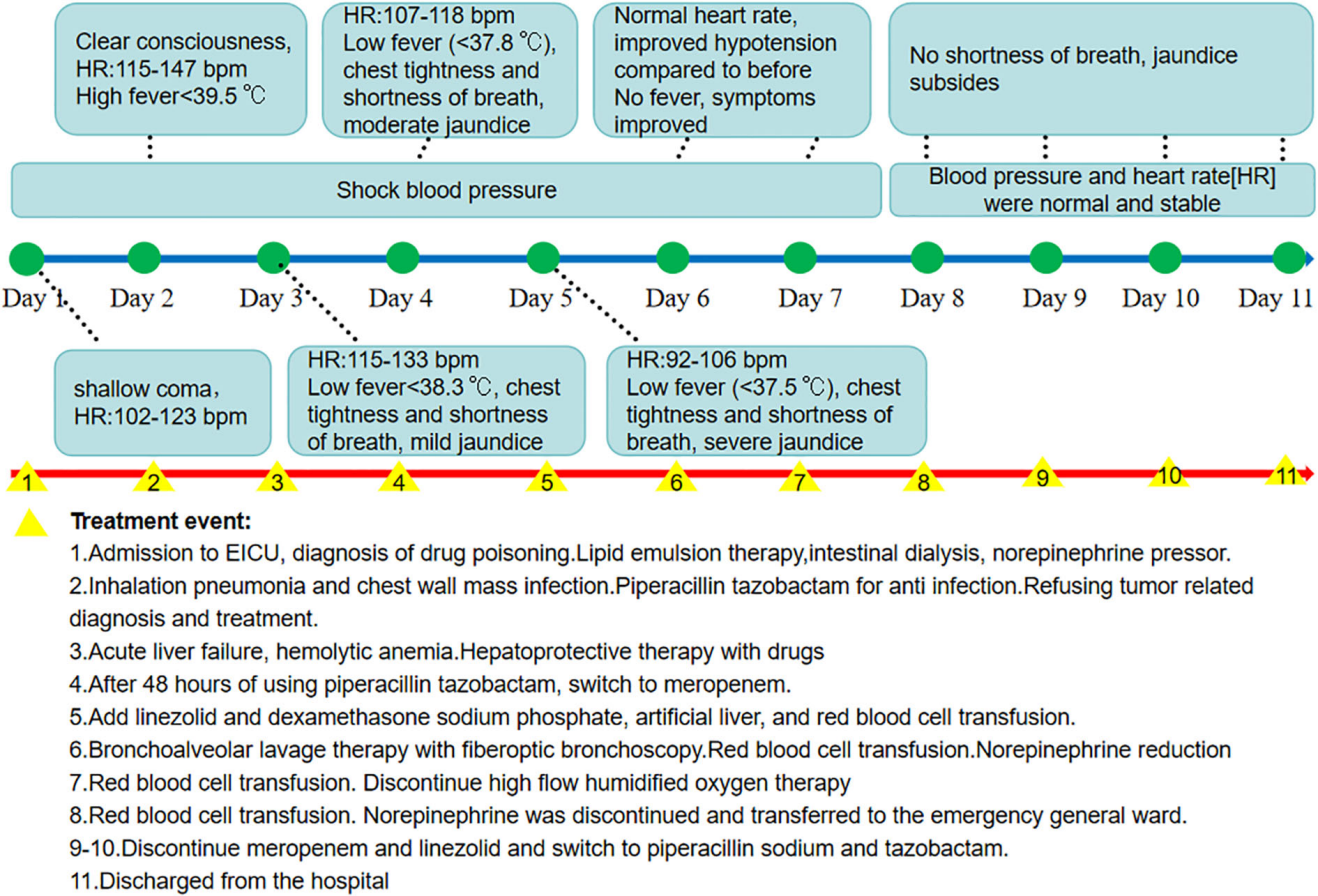
Timeline of clinical event development.

On the third day after admission, the patient experienced significant chest tightness and shortness of breath. The oxygenation index was below 200 mmHg, necessitating high-flow oxygen therapy via a ventilator. Enhanced CT of the chest and abdomen indicated: 1. A left breast mass suggesting breast cancer, with involvement of the left pectoralis major and minor muscles and significant lymph node metastasis in the left axilla and hepatic region.2. New infections in both lower lungs. 3. Multiple nodules in both lungs, obscured by consolidation. 4. Increased pleural effusion compared with previous scans. 5. Presence of gallstones and cholecystitis. Abdominal ultrasound showed no significant dilation of intrahepatic or extrahepatic bile ducts. Given the acute liver failure and lack of surgical intervention indications, treatment with adenosylmethionine, ursodeoxycholic acid, and reduced glutathione was initiated to protect the liver and reduce jaundice. The patient developed a fever upon admission. The fever peaked and then decreased, although inflammatory markers slightly increased. Sputum cultures revealed Staphylococcus aureus, indicating infection of the chest wall mass and worsening pulmonary infection. After 48 hours of piperacillin-tazobactam, treatment was switched to meropenem 1g Q8h. On the 5th day after admission, linezolid was added to strengthen Gram-positive coverage. Before the addition of linezolid, the patient had no fever, but hemoglobin progressively decreased from 126 g/L to 58 g/L. Tests for hepatitis virus markers, autoimmune liver disease antibodies, EB virus, cytomegalovirus, respiratory virus panel, and mycoplasma antibodies were all negative. The reticulocyte proportion was 4.17%, with a reticulocyte count of 0.1093 x 10^12/L, and peripheral blood showed anisocytosis and abnormal morphology, with evidence of compensatory hyperplasia in the bone marrow. Indirect bilirubin was significantly elevated. During the period of 5–8 days after admission, washed red blood cells (2U, 2U, 1U, 1.5U) were infused daily. From the 5th day of admission to discharge, dexamethasone sodium phosphate 10mg/day was intravenously injected. On the 5th day of admission, artificial liver was used, and plasma exchange combined with bilirubin adsorption was performed for 5.5 hours, resulting in the exchange of 2800mL of plasma. On the 6th day of admission, bronchoscopy revealed a small amount of yellow secretion in both lower lungs. Pathogen detection indicated the presence of hemolytic streptococcus in bronchoalveolar lavage fluid and a large amount of filamentous bacilli in the chest wall mass secretions. Following artificial liver treatment, liver function injury markers significantly decreased, with mild rebound in liver function indicators after 8 hours, but thereafter liver function steadily improved, with all blood test indicators showing improvement. Follow-up CT indicated partial absorption of pulmonary infection lesions, with no significant abnormalities in coagulation or renal function. Quetiapine levels fell below the detection limit. Anemia improved, and symptoms of chest tightness and shortness of breath resolved. Heart rate and blood pressure returned to normal, and jaundice disappeared. The patient was transferred out of the EICU on the 8th day of hospitalization and discharged on the 11th day as his condition improved. The timeline of clinical event development is shown in **Figure 4**.

## 3 Discussion

Aripiprazole and quetiapine are widely used in psychiatry. They are favored because of their safety, good tolerability, and fewer adverse effects compared to classical antipsychotics. Both drugs are highly lipophilic, have a large apparent volume of distribution, and are well absorbed when taken orally. According to the drug database data, the predicted log P values for the octanol/water partition coefficients of aripiprazole and quetiapine are approximately 3.76 and 2.81, respectively (25, 26). Aripiprazole has a bioavailability of 87% and a half-life of 72 hours. Its active metabolite has a half-life of approximately 94 hours. The drug exhibits linear pharmacokinetics within the 5–30 mg dosage range (8, 9). It is primarily metabolized in the liver via the cytochrome P450(CYP) system, specifically CYP2D6 and CYP3A4. CYP2D6 activity exhibits genetic polymorphism, which leads to significant interindividual differences in metabolic rates. These differences can affect the severity and duration of toxicity (10). Research on aripiprazole toxicity lacks large-scale clinical data; most existing reports are case studies of acute poisoning. Overdose can lead to several serious symptoms, including coma, seizures, and arrhythmias. Other possible effects include respiratory depression, hypotension, malignant syndrome, aspiration pneumonia, and extrapyramidal symptoms (9, 11–14). Quetiapine pharmacokinetics show peak blood concentrations reached 1.0-1.5 hours post-oral administration, steady-state concentrations achieved at 48 hours, with a protein binding rate of 83%. It is primarily metabolized by CYP3A4 into the active metabolite N-desalkylquetiapine. The elimination half-life is 7 hours for quetiapine and 12 hours for the metabolite (8). Quetiapine toxicity typically presents with central nervous system symptoms such as coma and seizures, and cardiovascular symptoms including hypotension and tachycardia. Other manifestations may include hyperprolactinemia, neuroleptic malignant syndrome, and in severe cases, multi-organ failure (15–17). There are no specific antidotes for toxicity from either drug.

In this case, the patient presented with mild coma, stable oxygen saturation, sinus tachycardia, and hypotension. He also showed mild dysfunction of the liver, kidneys, and heart. Additionally, the patient had soft tissue infection of the chest wall, aspiration pneumonia, and left breast cancer with axillary and liver metastases. The current international consensus guidelines clearly state that the effective therapeutic plasma concentration range for quetiapine is 100–500 ng/mL, and the therapeutic plasma concentration range for aripiprazole is 150–500 ng/mL (24). Based on symptoms and toxicology results, quetiapine toxicity was confirmed as the primary cause, with concentrations that exceeded reference values by 31.4 times and aripiprazole by 2.8 times. The patient regained consciousness by the second day of treatment but developed acute liver failure on the third day. Despite escalation and adjustment of antibiotic therapy, the timing of antibiotic use did not align with key changes in the patient’s condition. This suggests that severe infection was not the direct cause of liver failure. There was also no evidence of bone marrow suppression or antibiotic-related hemolysis. Throughout the course of the illness, the impact of antibiotics on heart rate and blood pressure was not significant. Instead, after artificial liver and steroid treatment, the patient’s hypotension and tachycardia improved, and inflammatory markers and liver function improved simultaneously. After the patient’s fever is controlled and inflammatory markers decrease, the patient still requires a small dose of norepinephrine to maintain blood pressure. Considering the results of laboratory tests including Chest CT examination, Bronchoscopy, hepatitis virus markers, autoimmune liver disease antibodies, other viral nucleic acids, and antigen detection, the disease does not meet the diagnosis of ARDS and septic shock, and is considered to be caused by drug poisoning leading to hypotension, acute liver failure, aspiration pneumonia, and hemolytic anemia. Although drug-induced acute liver failure is rare, the degree of liver injury caused by quetiapine is generally mild (1, 18). However, there have been reports of fatal acute liver failure due to quetiapine toxicity (6, 7). The patient refused tumor biopsy and anti-tumor treatment, and alpha-fetoprotein was negative, but based on enhanced CT characteristics, clinical diagnosis of liver metastasis of breast malignant tumor was made. Before admission, the patient’s liver function test was normal, but liver function failure occurred in a short period of time. Without anti-tumor treatment, liver function improved significantly within a few days, indicating that drug-induced liver failure is the dominant factor. Multiple literature reports indicate that quetiapine is more commonly associated with liver function impairment compared to aripiprazole, which corresponds with the toxic concentrations detected in this case. In patients with underlying liver disease, the clearance rate of drugs is lower (by 30%), necessitating cautious use and dose adjustments, along with enhanced monitoring of liver function. Current theories suggest that enhanced drug toxicity occurs through several pathways. These include inhibition of drug conversion to non-toxic metabolites, promotion of conversion to toxic metabolites, and saturation of alternative detoxification pathways. Together, these processes lead to hepatotoxicity (1). The patient’s drug-related liver failure is likely related to mixed drug toxicity and pharmacokinetic characteristics. In cases of poisoning where drugs reach steady-state, the amount cleared by blood purification may be limited. Reports (19) have indicated that patients rapidly clear quetiapine through blood perfusion (65% clearance within 12 hours), but it has also been found that quetiapine toxicity does not conform to one-compartment pharmacokinetics and may follow two- or three-compartment kinetics. Peak blood concentrations do not necessarily indicate peak concentrations in target organs, and during blood purification treatment, it is necessary to consider the re-entry of drugs from tissue distribution back into the bloodstream, which can cause symptom rebound. In this case, the patient’s consciousness changed from coma to consciousness after treatment with CRRT and fat emulsion, and the concentration of quetiapine significantly decreased before rebounding again. According to the existing lipid shuttle theory (27, 28), lipid emulsions may isolate lipophilic quetiapine from the central nervous system, and then lipid drug complexes may be transported to the liver for detoxification, leading to a decrease in plasma quetiapine levels. However, the half-life of triglycerides in fat emulsions is very short (13.7 ± 5.2 minutes), and the lack of sustained lipid shuttle activity may lead to an increase in serum quetiapine concentration rebound. It is necessary to extend fat emulsion treatment and increase the time and frequency of hemodialysis combined with hemoperfusion treatment after the patient’s consciousness is restored. In addition, in this case, the patient exhibited accelerated red blood cell destruction, rapid progressive decline in hemoglobin, significant elevation of indirect bilirubin and lactate dehydrogenase; significant increase in reticulocyte proportion and count, abnormal red blood cell morphology on peripheral blood smear, and clear evidence of compensatory hyperplasia on bone marrow examination. The patient had no history of active bleeding, and despite negative Coombs test, hepatitis virus, and autoimmune liver disease antibodies, these findings support the diagnosis of hemolytic anemia. Before and after the occurrence of acute liver failure and hemolytic anemia, the use of antibiotics did not have a malignant impact on the condition, preliminarily ruling out the toxic effects related to antibiotic drugs. Existing studies indicate that quetiapine can cause mitochondrial dysfunction, while therapeutic doses of aripiprazole show significant antioxidant protective effects under acute oxidative stress conditions *in vitro* (20). *In vitro*, it can induce sustained mitochondrial hyperpolarization and reactive oxygen species(ROS)production, and cause mild human red blood cell hemolysis (29). Moreover, there have been literature reports that quetiapine can induce thrombotic thrombocytopenic purpura (21, 22), and currently, there is the first reported case of autoimmune hemolytic anemia in a pediatric patient due to quetiapine use (23). We speculate that the combined toxicity of the two drugs may lead to intracellular ROS levels far exceeding those caused by a single drug, resulting in rapid depletion of glutathione (GSH), triggering hepatocyte apoptosis/necrosis and red blood cell hemolysis. Specifically, the accumulation of active metabolites after CYP3A4 saturation and the role of drug-induced oxidative stress in glutathione depletion leading to hemolytic anemia require further research. In summary, the acute liver failure and hemolytic anemia in this case are the result of multiple factors, but severe quetiapine (and aripiprazole) poisoning is the core driving factor. Liver metastasis from breast cancer reduced the metabolic reserve of the liver, and the systemic inflammatory response induced by chest wall and lung infection exacerbated the oxidative stress state, collectively constituting the “vulnerable” background for the patient’s susceptibility to drug toxicity. This case has limitations. We could not re-test aripiprazole concentrations during treatment because of toxicology testing constraints and the patient’s financial situation. Additionally, liver pathology biopsy was not performed, which prevented clarifying the tumor’s role in disease progression. Further etiological investigations and mechanistic studies on hemolytic anemia were also not conducted. The differential diagnosis analysis of potential causes of acute liver failure and hemolytic anemia is shown in **Table 1**.

**Table 1:** Differential diagnosis analysis of potential causes of acute liver failure and hemolytic anemia.

Potential etiology	Supporting evidence/refuting arguments for acute liver failure	Supporting evidence/refuting arguments for hemolytic anemia	Causality assessment
Quetiapine/ Aripiprazole Mixed Overdose	Supporting: ① Quetiapine blood concentration 31.4 times the reference value; ② Published literature reports quetiapine-associated acute liver failure; ③ Temporal relationship: liver failure occurred on day 3 post-overdose; ④ Liver function improved after artificial liver support therapy.	Supporting: ① Literature suggests quetiapine can induce hematological adverse reactions, with case reports indicating possible association with autoimmune hemolytic anemia; ② Temporal relationship with acute overdose event.	Highly Likely Primary Cause (Liver Failure)/Possible Trigger (Hemolysis)
Hepatic Metastases from Breast Cancer	Supporting: Contrast-enhanced CT indicated multiple liver metastases. Refuting: ① Normal liver function prior to admission; rapid deterioration of tumor burden within 2 days is unlikely; ② Liver failure pattern featured rapidly progressive hyperbilirubinemia, whereas metastatic disease typically first involves enzyme abnormalities; ③ No intrahepatic or extrahepatic bile duct dilatation, ruling out obstruction; ④ Liver function improved despite no antitumor treatment.	Supporting: Advanced malignancy can be associated with microangiopathic hemolytic anemia or anemia of chronic disease. Refuting: Anemia was acute, progressive, with clear hemolytic features (elevated indirect bilirubin, significantly increased reticulocytes), inconsistent with chronic cancer-related anemia.	Underlying Condition, but Not Direct Cause
Severe Infection (Chest Wall/Pulmonary)	Supporting: Presence of infectious foci, elevated inflammatory markers. Refuting: ① Liver failure occurred while infection markers were declining after antibiotic escalation; ② Hemodynamics inconsistent with septic shock (required vasopressors despite stable blood pressure); ③ Atypical presentation for sepsis-associated liver injury.	Supporting: Severe infection can cause hemolysis (e.g., toxin-producing strains). Refuting: ① No specific hemolytic bacteria identified in microbiological studies; ② Anemia progressed despite infection control; ③ Hemolysis occurred during antibiotic therapy.	Not Primary Cause
Multiple Antibiotics (Piperacillin-Tazobactam/Meropenem/Linezolid)	Supporting: Multiple antibiotics carry potential hepatotoxicity risks. Refuting: ① Liver failure occurred early during antibiotic therapy, timing inconsistent with drug exposure; ② Liver function did not immediately reverse upon antibiotic discontinuation/change; ③ Artificial liver support therapy was effective, implicating toxin removal rather than antibiotic cessation as the key factor.	Supporting: Antibiotics are a common cause of drug-induced hemolytic anemia (e.g., penicillins, linezolid). Refuting: ① Coombs test negative; ② Anemia was progressive before linezolid initiation; ③ Anemia improved with corticosteroid therapy while antibiotics were continued.	Low Likelihood
Cholecystitis/ Cholelithiasis	Supporting: Abdominal ultrasound suggested possible cholecystitis. Refuting: ① No intrahepatic or extrahepatic bile duct dilatation, no evidence of obstructive jaundice; ② Liver failure pattern was hepatocellular (elevated enzymes) rather than obstructive (isolated hyperbilirubinemia); ③ Absence of typical right upper quadrant tenderness.	Not supported: No clear association with hemolysis.	Can Be Excluded

## 4 Conclusion

Patients with underlying liver disease should avoid combined use of antipsychotic medications, implement precise medication management, and strengthen liver function monitoring. When drug toxicity occurs, it may be necessary and effective to initiate extended blood dialysis combined with blood perfusion treatment early to clear toxins. Quetiapine may cause drug-induced hemolytic anemia. Treatments such as artificial liver support—including plasma exchange and bilirubin adsorption—washed red blood cells transfusion, and anti-inflammatory steroids can significantly improve outcomes in liver failure and hemolytic anemia.

## Patient perspective

Despite the clinical improvement, the patient still showed anxiety and concern about financial expenses, with a relatively low mood and a negative attitude towards treatment throughout the course of the disease.

## Data Availability

The datasets presented in this study can be found in online repositories. The names of the repository/repositories and accession number(s) can be found in the article/**Supplementary Material**.
