# Supplementary material for: Acute liver failure and hemolytic anemia induced by quetiapine and aripiprazole overdose in a patient with schizophrenia and metastatic breast cancer: a unique case report
Source: Front Psychiatry. 2026 Apr 13;17:1782761. doi: 10.3389/fpsyt.2026.1782761 (PMC13111436; doi:10.3389/fpsyt.2026.1782761)
Supplement: Supplementary file 1 [file DataSheet1.docx]

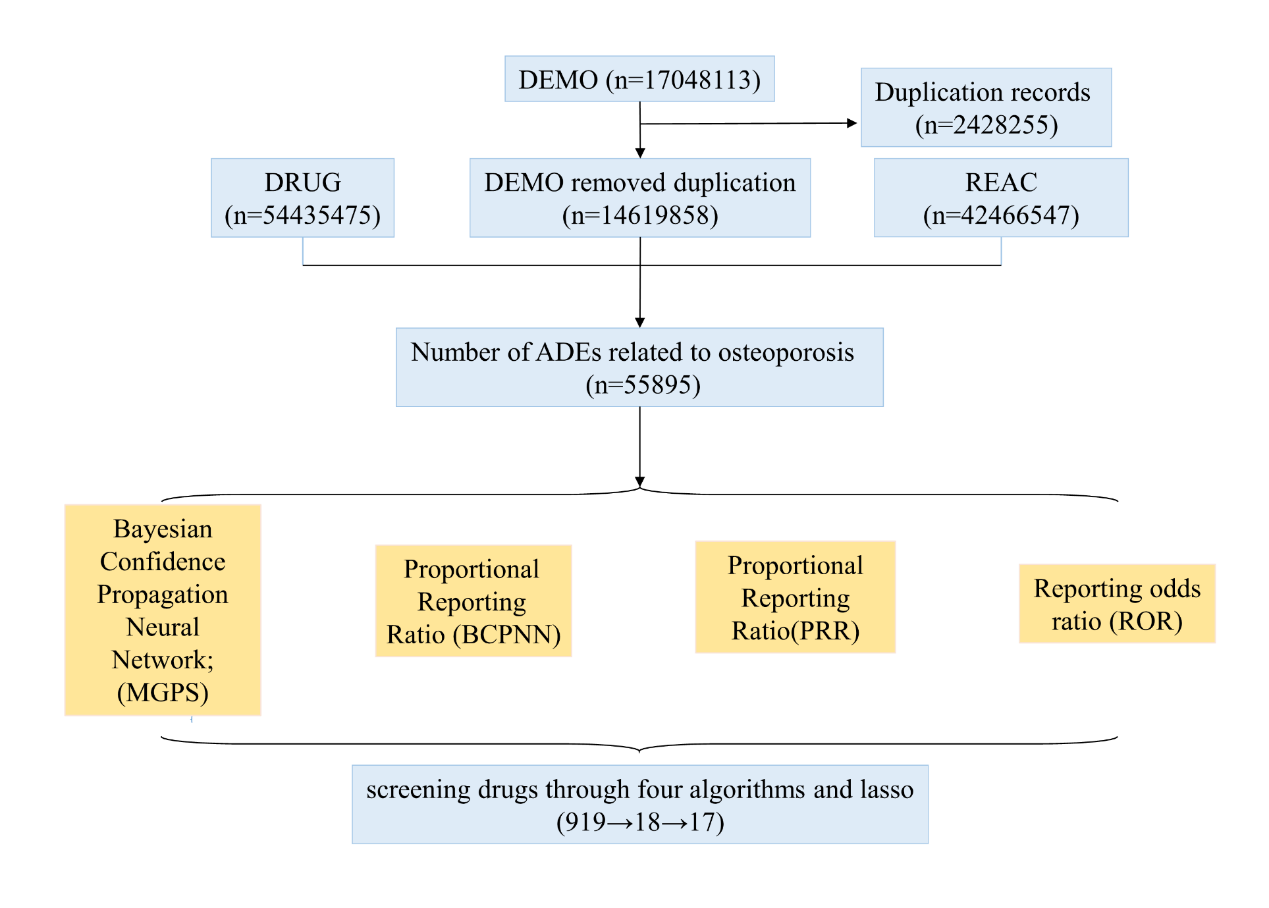


Supplementary Figure 1: Flowchart of drug selection for osteoporosis-related adverse events.

| **Abbreviation** |  |
| --- | --- |
| WHO | World Health Organization |
| DOP | Drug-induced osteoporosis |
| FAERS | FDA Adverse Event Reporting System |
| LASSO | Least Absolute Shrinkage and Selection Operator |
| DEMO | Demographic and administrative information |
| REAC | MedDRA-coded adverse events |
| DRUG | Reported drugs and biological products |
| OUTC | Patient outcomes |
| RPSR | Report sources |
| THER | Drug therapy start and end dates |
| INDI | MedDRA-coded indications |
| PTs | Preferred terms |
| ROR | Odds ratio |
| PRR | Proportional reporting ratio |
| BCPNN | Bayesian Confidence Propagation Neural Network |
| MGPS | Multi-Item Gamma Poisson Shrinker |
| ADR | Adverse drug reaction |
| OT | Other serious outcome |
| HO | Hospitalization |
| NRTI | Nucleoside reverse transcriptase inhibitor |
| RANKL | Receptor activator of nuclear factor-κB ligand |
| OPG | Osteoprotegerin |
